# Utility of the BioFire^®^ FilmArray^®^ Pneumonia Panel *plus* assay for syndromic testing of lower respiratory tract infections in a low/middle-income setting

**DOI:** 10.1093/jacamr/dlac139

**Published:** 2023-01-04

**Authors:** M Van Der Westhuyzen, N Samodien, A J Brink, C Moodley

**Affiliations:** Division of Medical Microbiology, Faculty of Health Sciences, University of Cape Town, Cape Town, South Africa; National Health Laboratory Service, Microbiology, Groote Schuur Hospital, Cape Town, South Africa; Division of Medical Microbiology, Faculty of Health Sciences, University of Cape Town, Cape Town, South Africa; National Health Laboratory Service, Microbiology, Groote Schuur Hospital, Cape Town, South Africa; Division of Medical Microbiology, Faculty of Health Sciences, University of Cape Town, Cape Town, South Africa; National Health Laboratory Service, Microbiology, Groote Schuur Hospital, Cape Town, South Africa; Division of Medical Microbiology, Faculty of Health Sciences, University of Cape Town, Cape Town, South Africa; National Health Laboratory Service, Microbiology, Groote Schuur Hospital, Cape Town, South Africa

## Abstract

**Background:**

Determining lower respiratory tract infection (LRTI) aetiology is complex. Culture-based methods are laborious with poor sensitivity. Molecular assays improve detection of potential pathogens, but incorrect interpretation of results may lead to inappropriate antimicrobial therapy.

**Methods:**

The utility of the BioFire^®^ FilmArray^®^ Pneumonia Panel *plus* (FA-PP) to detect LRTI pathogens, and the potential impact on antimicrobial stewardship in a low-resource setting, were assessed. Routine LRT samples were included from adult patients with clinically suspected LRTI or with a concomitant blood culture at Groote Schuur Hospital and referring facilities. Culture and FA-PP results were compared, and pharmacy data analysed to determine appropriateness of antibiotic therapy.

**Results:**

There was an 80% correlation between cultured LRTI pathogens and the FA-PP bin ≥10^7^ results. Compared with culture, the FA-PP detected substantially more pathogens (86.6% versus 17.9%) and produced a combined 100% positive percent agreement, and 88% negative percent agreement. The FA-PP detected bacterial/viral coinfections in 27% of samples. Correlation of FA-PP results with pharmacy data (*n* = 69) indicated a potential antibiotic change in 75% of cases, but this is difficult to accurately characterize without a ‘gold standard’ for treatment or complete clinical data.

**Conclusions:**

The FA-PP increased the number of positive samples with typical bacteria, but the semi-quantitative reporting algorithm does not describe the correlation between the different bin values and colonization versus infection. This complicates result interpretation and may lead to inappropriate antimicrobial treatment. This study highlights the potential positive impact of rapid molecular assays for routine care in lower-income settings, but also underscores the interpretive challenges associated with these tests.

## Introduction

Antibiotics are the cornerstone for pneumonia therapy and target the most common bacterial causes. It is often difficult to discern clinically and/or radiologically whether the cause of the pneumonia is viral, bacterial, or even non-infectious.^1^ In the setting of community-acquired pneumonia (CAP) there is emerging evidence that viruses play a greater role than initially considered and implies that antibiotics may be unnecessarily prescribed in many instances.^1^ Overprescribing of broad-spectrum antibiotics also occurs in the hospital setting where up to 50% of in-hospital patients may not have bacterial pneumonia.^2^ This indiscriminate use of antibiotics may promote the development or selection of antimicrobial resistance (AMR), one of the leading WHO global health threats.^3^

Identifying the bacterial aetiology of lower respiratory tract infection (LRTI) in clinical respiratory samples using conventional culture methods is laborious and can take up to 5 days to finalize complex cultures and determine susceptibility profiles.^4^ Implementing antimicrobial stewardship (AMS) principles in these settings is therefore challenging, and broad-spectrum antibiotics are generally continued until conventional microbiology results are available.^4,5^ Various factors contribute to the sensitivity and turnaround times of conventional cultures. These include failure to isolate the aetiological agent due to antimicrobial exposure prior to obtaining the sample, overgrowth of contaminating or normal microbiota, which may mask the pathogen, as well as failure of routine culture media to support the organism-specific growth requirements of atypical bacteria.^6,7^

Molecular methods have become an attractive alternative to conventional culture methods to detect bacterial pathogens. These tests can overcome the limitations of conventional microbiological methods by providing rapid and more sensitive results without the need for viable organisms. This may assist in making clinical decisions earlier, improve patient outcomes, and ultimately reduce the unnecessary use of antimicrobial agents.^5,8–10^

One such syndrome-specific system, which has been evaluated in numerous studies, is the BioFire^®^ FilmArray^®^ Pneumonia Panel *plus* (FA-PP; bioMérieux, Marcy l’Étoile, France). It is a rapid, cartridge-based, multiplex PCR assay detecting nucleic acids from various bacterial and viral respiratory pathogens causing pneumonia, as well as common AMR genes. This assay is approved for respiratory samples such as sputum, endotracheal aspirate (TA) and bronchoalveolar lavage (BAL) samples,^11^ and is US FDA-cleared, CE-marked for *in vitro* diagnostic medical devices and Therapeutic Goods Administration-certified for diagnostic use.

This assay includes 15 typical pneumonia-causing bacteria (typical bacteria), 9 viruses, 3 atypical bacteria, as well as AMR genes for MDR organisms such as ESBL- and carbapenemase-producing organisms and MRSA. The presence of AMR genes is reported qualitatively, but only when an associated bacterium is simultaneously detected by the panel.

Development of commercial multiplex panels for the diagnosis of LRTI is rapidly progressing, but limited data are available to guide informed clinical decision-making regarding the utility of rapid diagnostic tests (RDTs) in low/middle-income countries (LMICs). To address this gap, this single-centre, cross-sectional pilot study was performed, determining the diagnostic utility of the BioFire^®^ FA-PP panel for detecting LRTIs to assess the potential AMS impact, pertaining to organism identification and/or mechanism of resistance.

## Methods

### Design

A single-centre, cross-sectional, laboratory diagnostic study comparing the results of conventional microbiological investigations of LRTI specimens with that of the BioFire^®^ FA-PP panel.

### Setting

The study was conducted at the National Health Laboratory Services (NHLS), Microbiology Laboratory, C18, located at Groote Schuur Hospital (GSH), Cape Town, South Africa. GSH provides tertiary and quaternary care for a large population in the City of Cape Town Metropolitan, and both the hospital and the on-site NHLS laboratory serve as a referral centre for regional and district hospitals.

### Ethics

Ethics approval for this work was granted by the University of Cape Town Human Research Ethics Committee (HREC reference number: 769/2020) and a waiver of the requirement for informed consent was obtained.

### Study population

The study population included adult (≥18 years) inpatients and outpatients at GSH or any of the referring healthcare facilities. Samples were collected between 24 February and 3 April 2021 using a convenience sampling method. All LRTI (47 tracheal aspirates and 78 sputum) samples submitted to the NHLS laboratory by the treating clinician as part of routine patient care for routine microbiological investigations were considered for inclusion. Respiratory samples were included in the study if they included clinical suspicion of an LRTI as stated on the laboratory request form. Since the suspicion of LRTI is rarely indicated on laboratory request forms, this study included all respiratory tract samples without a recorded clinical indication, where a blood culture was submitted within 2 days of the respiratory tract sample, to serve as a proxy for sepsis attributable to severe respiratory infection. All selected samples had to be of sufficient volume to perform standard diagnostic tests and additional tests requested by the treating clinician, with a minimum of 500 μL residual sample for the FA-PP assay. Duplicate samples were only included if a previous sample from the same patient had been included >10 days prior.

### Sample size

Using the MKmisc R package^12^ to run the power diagnostic test script with the selected criteria of α = 0.05, power = 0.8 and δ = 0.1, an approximate sample size of 136 specimens was required to determine the positive percent agreement (PPA) and negative percent agreement (NPA) assuming 95% sensitivity for the FA-PP assay based on published literature and an expected prevalence of detectable pathogens in 50% of selected samples.

### Laboratory procedures

LRT samples that met the inclusion criteria were processed within 48 h of sample receipt at the laboratory. Routine microbiological investigations included Gram stain microscopy, culture and susceptibility testing (MC&S). The quality of sputum samples was assessed using the Bartlett scoring system, and samples with a score <1 were excluded from being processed for culture.^13^ For semi-quantitative culture, chocolate blood agar, Colgent (Columbia blood agar with gentamicin) with the addition of a 10 μg/mL optochin disc, and a MacConkey agar plate (MCC) were inoculated and incubated in a carbon dioxide incubator, with the MCC agar plate aerobically, both at 35°C for 24 h. Respiratory pathogens were identified and graded by trained laboratory technologists and followed up according to standard operating procedures. The VITEK 2 instrument (bioMérieux, Marcy l'Étoile, France) was used to identify any bacterial growth deemed significant, and their susceptibility profiles determined and interpreted using the CLSI 2021 guidelines.^14^

The FA-PP assay was performed according to the manufacturer’s instructions using 200 μL of the residual sample once all routine laboratory testing was complete. The results of the FA-PP assay and routine laboratory testing were recorded by the investigator once all tests were finalized. FA-PP assay results were not communicated to the laboratory staff to prevent bias and the investigator only informed the treating clinicians of notifiable organisms detected.

### Data collection and analysis

The results obtained from routine laboratory testing and the FA-PP assay, as well as basic patient demographics (age, gender, hospital, ward) available on the laboratory information system (TrakCare), and the electronic prescribing data obtained from the pharmacy, were captured in a Microsoft Excel spreadsheet. Descriptive statistical analyses were performed using Microsoft Excel and Stata software version 17.0 (StataCorp, College Station, TX, USA).

The FA-PP assay is able to detect common bacterial pathogens causing pneumonia and these were classified as either ‘typical’ or ‘atypical’. The presence and absence of each typical bacterial target as detected by the two methods were collated and the PPA and NPA were calculated for each bacterial target using GraphPad Prism version 9.3.1.

The FA-PP assay reports typical bacteria semi-quantitatively and this function is based on a binning algorithm where the relative number of PCR amplicons is measured and compared with an internal standard curve. Bins are reported as 10^4^, 10^5^, 10^6^ or ≥10^7^ copies/mL, and each bin represents a density range of about 1 log unit, with upper and lower limits, for example: the 10^4^ bin is equivalent to 10^3.5^ to 10^4.5^ copies/mL.^8,11^

The detection of AMR genes was compared with the phenotypic susceptibility test results obtained from the VITEK 2. Atypical bacteria in the FA-PP panel are not routinely tested for in the diagnostic laboratory and were therefore not compared with other methods.

Pharmacy records of included patients, where available, were reviewed by a clinical microbiologist to assess whether the prescribed antibiotic was appropriate for the FA-PP pathogens detected, based on current clinical microbiological practices. After completion of the FA-PP assay, and due to incomplete clinical data, the following assumptions were made for this analysis: (1) only typical bacteria detected in bin ≥10^7^ were considered significant; (2) all atypical bacteria detected on the FA-PP assay were regarded as significant; (3) resistance genes detected were only considered significant if a corresponding typical organism was also detected at bin ≥10^7^; (4) all antibacterial agents issued on the same day or within 2 days of sample collection were assessed for appropriateness; (5) if no antibiotics were issued on the same day or within 2 days of sample collection, antibacterial agents prescribed prior to sample collection were assessed, but only if sufficient doses were prescribed to overlap the sample collection day; (6) all antibacterial agents were prescribed for an LRTI. The bin ≥10^7^ value was considered significant as previous studies reported the overestimation of quantification using the FA-PP assay, when compared with culture.^7,15,16^ Additionally more than 85% of bacteria considered as significant by culture were also reported with a bin ≥10^6^ or bin ≥10^7^ by Gastli *et al.*^17^ and Yoo *et al.*,^6^ respectively. The potential impact on patient treatment, using the results obtained using the FA-PP assay, were classified as ‘no change’, ‘escalation’, ‘de-escalation’ or ‘discontinuation’.

## Results

A total of 125 LRT samples (47 TAs and 78 sputum samples) were collected during the 5 week study period. The samples were collected from 123 patients with suspected LRTI, admitted or treated as an outpatient at GSH and from 11 surrounding referral hospitals. Most samples were collected from ICU patients, followed by general ward patients (Table 1).

**Table 1.** Sample and patient characteristics

	Number (n = 125)	Percentage
Sample type		
Sputum	78	62.4
TA	47	37.6
Ward type		
ICU	47	37.4
General ward	43	34.4
Emergency centre	22	17.6
Outpatient clinic	3	2.4
Not indicated	10	8.0
Gender		
Male	80	64.0
Female	45	36.0
Age, years (range)		
Combined median	47 (18–90)	
Male median	47 (18–90)	
Female median	45 (18–81)	

**Figure 1.**
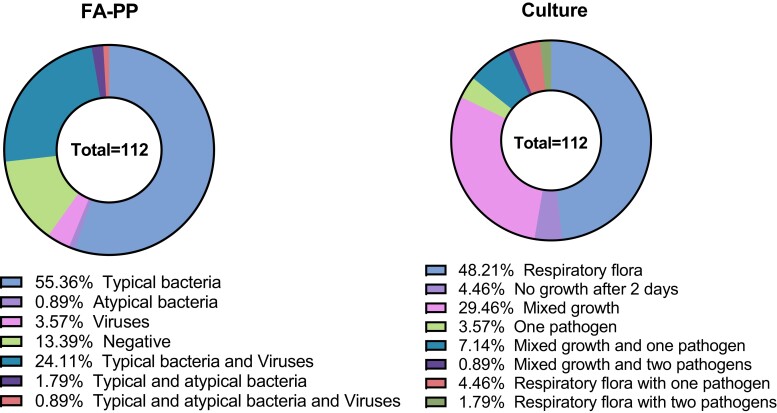
Proportions of pathogens detected using the FA-PP and routine laboratory testing.

Of the 125 samples collected, 13 were rejected for further routine testing based on the Bartlett test results. The FA-PP assay detected pathogens in 86.6% (97/112) of the samples that were tested with both methods, and this included typical bacteria in 55.4% (62/112), atypical bacteria in 0.9% (1/112), viruses in 3.6% (4/112) and coinfections in 26.8% (30/112) of the samples. Routine culture only detected pathogens in 17.9% (20/112) of the samples tested (Figure 1).

Organisms not included in the FA-PP assay panel were detected in three samples using routine culture, including *Aeromonas hydrophila*, *Corynebacterium striatum* and *Morganella morganii*. The most commonly detected pathogen using both methods was *Haemophilus influenzae*, with 38.4% of samples using the FA-PP assay, and in 4.5% samples with routine testing. The second most common pathogen detected by the FA-PP was *Staphylococcus aureus*, while the second most common pathogens detected by culture were both *Streptococcus pneumoniae* and *Pseudomonas aeruginosa* (Table 2).

**Table 2.** Typical and atypical bacteria detected, with PPAs and NPAs

Typical bacteria	Culture (n)	FA-PP (n)	PPA (95% CI)	NPA (95% CI)
*H. influenzae*	5	43	100 (57–100)	64 (55–73)
*S. aureus*	1	30	100 (5–100)	74 (65–81)
*S. pneumoniae*	4	28	100 (51–100)	78 (69–85)
*Klebsiella pneumoniae*	1	27	100 (5–100)	77 (68–83)
*Enterobacter cloacae complex*	2	18	100 (18–100)	85 (78–91)
*Acinetobacter calcoaceticus-baumannii complex*	2	16	100 (18–100)	87 (80–92)
*P. aeruginosa*	4	11	100 (51–100)	94 (87–97)
*Escherichia coli*	0	10	—	91 (84–95)
*Moraxella catarrhalis*	0	9	—	92 (85–96)
*Proteus species*	0	6	—	95 (89–98)
*Klebsiella oxytoca*	0	4	—	96 (91–99)
*Serratia marcescens*	1	4	100 (5–100)	97 (92–99)
*Klebsiella aerogenes*	0	3	—	97 (92–99)
*Streptococcus agalactiae*	0	3	—	97 (92–99)
*Streptococcus pyogenes*	0	2	—	98 (94–100)
*Total*	20	214	100 (84–100)	88 (87–90)
Atypical bacteria	Culture (n)	FA-PP (n)		
*L. pneumophila*	4^a^	4		
*Chlamydia pneumoniae*	N/A	0		
*Mycoplasma pneumoniae*	N/A	0		
*Total*		4		
Viral pathogens	PCR (n)	FA-PP (n)		
Human rhinovirus/enterovirus	N/A	19		
Parainfluenza virus	N/A	5		
Coronavirus	N/A	4		
Respiratory syncytial virus (RSV)	N/A	4		
Adenovirus	N/A	2		
Human metapneumovirus	N/A	0		
Influenza A	N/A	0		
Influenza B	N/A	0		
Middle East respiratory syndrome coronavirus (MERS)	N/A	0		
Total		34		
Resistance genes	Phenotypic (n)	FA-PP (n)		
CTX-M	3^b,c^	16		
mecA/C and MREJ	0	6		
NDM	2^c,d^	5		
OXA-48-like	0	3		
IMP	0	0		
KPC	0	0		
VIM	0	0		
Total	5	30		

N/A, not applicable.

a Two out of the four L. pneumophila detections confirmed with positive L. pneumophila serogroup 1 urinary antigen test, the rest confirmed with a PCR test for atypical pneumonia by the NICD as non-serogroup 1.

b Carbapenem-susceptible P. aeruginosa (WT) was detected by routine laboratory testing, but FA-PP detected CTX-M, NDM and OXA-48-like genes in the presence of Acinetobacter calcoaceticus-baumannii complex, K. pneumoniae and P. aeruginosa. Assumption made that P. aeruginosa only associated with CTX-M.

c Carbapenem-resistant A. baumannii detected by routine laboratory testing, but FA-PP detected CTX-M and NDM genes in the presence of Acinetobacter calcoaceticus-baumannii complex and K. pneumoniae. Assumption that A. baumannii associated with both genes.

d Carbapenem-resistant A. baumannii detected by routine laboratory testing, but FA-PP detected the NDM gene in the presence of Acinetobacter calcoaceticus-baumannii complex and P. aeruginosa. Assumption made that A. baumannii associated with the NDM gene.

The most commonly detected virus was human rhinovirus/enterovirus. No tests for respiratory viruses, except for SARS-CoV-2, were requested as part of routine care and thus no comparison between methods was possible. *Legionella pneumophila* was detected in four samples; for three of these patients a *Legionella* urinary antigen test (serogroup 1) was not requested as part of routine testing. A confirmatory *Legionella* urinary antigen test (serogroup 1) was performed by the investigator following discussion of the FA-PP results with the treating clinicians. Two of these samples tested negative for *L. pneumophila* serogroup 1 and were later confirmed by the reference laboratory (National Institute for Communicable Diseases, NICD) as *L. pneumophila* non-serogroup 1.

A total of 30 resistance genes were detected using the FA-PP assay. Of these, 22 samples had one gene detected, 1 sample had two genes, and 2 samples had three genes. The *bla*_CTX-M_ gene was detected most commonly (14.3%), followed by *mecA*/*C* (5.4%), *bla*_NDM_ (4.5%) and *bla*_OXA-48-like_ (2.7%).

The FA-PP detected pathogens in 11/13 sputum samples that were rejected for routine culture due to a low Bartlett score. This produced a total of 17 typical bacteria (ranging from 1 to 3 per sample) and 8 viral pathogens. Two samples also contained either the *bla*_CTX-M_ or *bla*_OXA-48_ resistance gene.

The qualitative assessment of the FA-PP assay to detect typical bacterial targets demonstrated a combined 100% PPA and 88% NPA compared with routine culture (Table 2). In 18/20 samples where bacteria were detected with both methods, the FA-PP reported a bin ≥10^7^ result. The concordance for semi-quantitation of the two methods was not determined due to the low number of pathogens detected by the routine laboratory testing.

**Figure 2.**
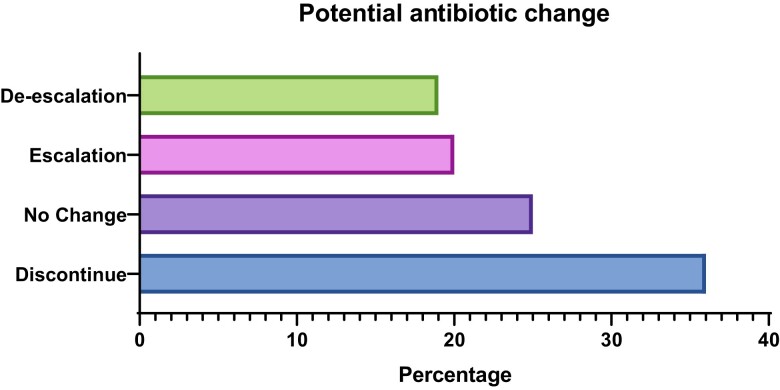
Potential antibiotic change based on FA-PP findings. De-escalation, narrower-spectrum agents (or fewer agents) indicated; escalation, broader-spectrum agents (or additional agents) indicated; no change, agents appropriate; discontinue, no targets detected, only viral pathogens detected or bacteria detected with a bin value of less than 10^7^.

Pharmacy data were available for 69/125 samples (67 participants) and in 75% (*n* = 52) of these cases a potential antibiotic change was possible based on the FA-PP assay results (Figure 2).

## Discussion

Compared with conventional microbiology, novel and rapid panel-based diagnostic strategies offer clear advantages of a shorter turnaround time, increased sensitivity and the detection of fastidious microorganisms, including AMR determinants, and may thus offer substantial improvements in patient care. Numerous studies report the performance and benefits of respiratory syndromic panels such as the FA-PP assay; however, most were performed in high-income countries using BAL specimens and samples collected from patients admitted to ICUs.^7,15,16,18^ Additionally, the impact on patient-level outcomes have yet to be determined.^19^

We aimed to assess the utility of the FA-PP assay using all routine LRT samples submitted to a South African laboratory for routine microbiological investigations. The samples included were collected at various healthcare settings in order to assess patients with a range of disease severities. More than half of the samples collected were sputum samples. The routine laboratory testing had a poor yield, where ≥1 pathogen/s were detected in less than a quarter of samples. More pathogens were detected in TA samples, but ‘mixed growth’ or ‘respiratory flora’ were reported in a large number of both TA and sputum samples, which is common for these sample types.

The poor diagnostic yield of sputum samples from patients with suspected CAP is well described and for this reason the collection is discouraged in many guidelines, including the South African guideline for managing CAP^20^ and ATS/IDSA,^21^ which indicate that MC&S has a limited impact on patient management and outcome and should only be requested in cases of severe disease and where there is a high risk for an infection with nosocomial pathogens.^21^ In contrast, the IDSA guidelines for the management of hospital-acquired pneumonia and ventilator-associated pneumonia promote the collection of non-invasive samples over invasive samples due to the lack of evidence that invasive sampling improves clinical outcome.^22^ Unfortunately, these non-invasive sample types are prone to contamination with upper respiratory tract commensals or colonizing microorganisms, especially in patients with chronic tracheostomies, where the tracheostomy tube is colonized.^8,10^ A BAL may theoretically provide a ‘superior’ quality result due to the site-directed collection^10^ limiting contamination, which may imply organisms cultured from these samples will most likely better reflect the true pathogen causing the LRTI, which further simplifies interpretation of the laboratory report. BAL is, however, not a realistic option for all patients due to limited resources in lower-income settings, as well as the invasiveness and complicated nature of specimen collection.

The use of the FA-PP assay increased the number of samples where typical bacteria were detected by 68.7%. This dramatic increase may appear as an increase in sensitivity over culture; however, we detected a concerning amount of additional typical bacteria, not detected with culture, using the FA-PP (194/214). A similarly increased positivity rate (63.3%) was also reported by Buchan *et al*.^7^ However, that study was much larger and assessed 259 BAL samples, but only reported 73 additional bacterial targets. This finding is likely attributed to the non-invasive sample types in our study, which probably contained more contaminating commensals.

The FA-PP assay produced a PPA of 100% for 8/15 of the typical bacteria detected by culture, which was comparable with other studies evaluating the FA-PP. Buchan *et al*.,^7^ Lee *et al*.,^16^ Ginocchio *et al*.,^15^ Mitton *et al*.,^23^ Weber *et al*.^18^ and Yoo *et al*.^6^ all reported a PPA (or sensitivities) for bacterial pathogens of ≥90%. The combined NPA was 88% for the typical bacteria, with all targets having an NPA ≥64%, which was lower than those reported in the above studies, except for Yoo *et al*.,^6^ who reported a specificity of 76.5%. Due to the lack of a ‘gold standard’ diagnostic test for LRTI these detections cannot, however, be described as false positives.^16^

Unfortunately, the interpretation of these additional detections using the FA-PP assay is complicated by a number of factors. PCR-based assays are more sensitive than routine culture and can detect genetic material of both viable and non-viable organisms. This is advantageous where antibiotics were administered prior to sample collection leading to some organisms being undetectable by culture. Since there were no records of antibiotics prior to sample collection or time of antibiotic administration for the participants in this study, no correlations could be made.

In cases where bacteria are cultured, the laboratory’s protocol will guide reporting. Bacterial isolates that are grown in insufficient quantities, mixed with more than two potential pathogens, or where the growth is overwhelmed by normal respiratory microbiota may not be regarded as significant, and therefore not reported. Bacteria such as *H. influenzae* are fastidious to culture and may easily be overgrown by normal microbiota or lose viability^15^ and may as a result be missed by culture. *H. influenzae* was the most common pathogen detected by both methods, but the FA-PP detected *H. influenzae* in 38 additional samples. The FA-PP also detected 29 additional samples with *S. aureus*. A similar finding was also described by Buchan *et al*.^7^ and Ginocchio *et al*.^15^

The quality of the sample is also vital to ensure that significant typical bacterial pathogens are not missed by routine laboratory testing. We included 13 sputum samples that were rejected for routine culture in order to assess the value of these samples for detecting potential pathogens. Typical bacteria were detected by the FA-PP assay in nine of these samples and this most likely reflects contamination or colonization in the absence of inflammation. This illustrates the importance of quality assessment prior to culture or molecular tests for typical bacteria, because neither of these methods are able to separate colonizers from invasive pathogens.^10^ However, quality scoring systems for sputum samples do not apply in LRTIs caused by atypical bacteria and viruses.^24^ In fact, viral pathogens were detected in eight of these samples that were rejected due to poor quality. This demonstrates that future studies need to assess the value of a quality scoring system for the FA-PP to enable optimal detection of all clinically relevant pathogens.

The semi-quantitative reporting of bacterial results using the FA-PP assay, which is intended to simplify this discrimination, lacks the ability to definitively interpret organisms that may be colonizers.^8^ Nearly all of the typical bacteria reported by the routine testing correlated with an FA-PP bin of ≥10^7^, but the majority of all the detected typical bacteria were also reported with a bin of ≥10^7^. In addition to the large number of typical bacteria that were detected per sample, a combination of bacterial and viral pathogens was also detected in 27% of samples. This further complicated the interpretation of results, since coinfections with bacterial and viral pathogens are possible, but the detection of a viral pathogen can also be due to asymptomatic carriage or a recent viral infection that led to a subsequent secondary bacterial infection.^25^

The rapid detection of AMR genes can lead to earlier escalation or de-escalation of therapy in patients, as well as earlier infection prevention and control interventions. Although the FA-PP assay detected resistance genes in some of the samples, the pathogen it was associated with was not always detected by culture and the results could therefore not be compared.

The assessment of potential impacts on antibiotic therapy using the FA-PP results was based on typical bacteria detected with a bin ≥10^7^ only, associated resistance genes, atypical bacteria and viruses, by applying local microbiology practices. In clinical practice, multiple factors must be considered before an antibiotic change is made. In this retrospective assessment with limited clinical information, we identified 52/69 cases where a potential antibiotic change was possible including de-escalation (19%), escalation (20%) and discontinuation of antibiotics (36%). This may have significant impacts on patient outcomes, AMS and infection prevention and control interventions.

As described by Hanson *et al*.,^10^ the enhanced detection of a multiplex nucleic acid amplification test decreases the likelihood that important pathogens are missed, but also complicates result interpretation and ultimately patient management. The use of the FA-PP assay on routine samples and the reporting of results without the input of a clinical microbiologist or infectious diseases specialist may lead to overtreatment due to the large number of additional bacterial detections with unclear clinical significance. Advocating the use of these diagnostic assays without firmly establishing criteria for which patients would benefit most, how to meaningfully interpret the results, and how to treat accordingly, could in fact be counterproductive with regard to diagnostic ‘best practice’ and AMS.

This study had several limitations, most importantly the lack of a ‘gold standard test’, which complicated the comparison of methods. The small sample size produced wide CIs for comparisons and no definite conclusions could be made. Complete records of clinical data would have improved the assessment of potential antibiotic changes; also, information on antimicrobial exposure prior to sample collection would have simplified interpretation of the results. We were also unable to compare the cost differential between the methods, and therefore definitive recommendations as to the value-added benefit of introducing such an assay in our setting cannot be made.

Future work should focus on determining semi-quantitative values where relative pathogen abundance may assist in determining the significance of a specific pathogen. Similarly, the timing of the specimen in the course of disease may be a confounder and requires elucidation.

### Conclusions

Despite the limitations in our study, the FA-PP assay substantially increased the number of positive samples with typical bacteria. A potential antibiotic change was possible in 75% of cases. Our study highlights the potential impact of introducing rapid molecular assays in routine care in settings such as ours but underscores the interpretive challenges associated with novel rapid tests.

## Data Availability

The data that support the findings of this study are available from the corresponding author, M.V.D.W, upon reasonable request.
